# Editorial: Interplay between RNA-binding proteins and non-coding RNAs in tumor therapeutic resistance

**DOI:** 10.3389/fonc.2023.1201122

**Published:** 2023-04-20

**Authors:** Peixin Dong, Mohammad Taheri, Feng Wang

**Affiliations:** ^1^ Department of Obstetrics and Gynecology, Hokkaido University School of Medicine, Hokkaido University, Sapporo, Japan; ^2^ Institute of Human Genetics, Jena University Hospital, Jena, Germany; ^3^ Department of Laboratory Medicine, Affiliated Hospital of Nantong University, Nantong, China

**Keywords:** non-coding RNA, microRNA, long non-coding RNA, circular RNA, RNA binding protein, tumor biomarker, tumor therapeutic resistance

Resistance to chemotherapy, targeted therapy, and immunotherapy is the primary cause of treatment failure and death. RNA-binding proteins (RBPs) are a large class of proteins that interact with RNA molecules to control how they function. Among other RNA types, these proteins may bind to messenger RNA (mRNA), microRNA (miRNA), long non-coding RNA (lncRNA), and circular RNA (circRNA). The interplay between RBPs and RNA elements influences RNA processing, stability, transport, and translation, as well as RNA splicing, editing, and degradation. RBPs also play a crucial role in gene expression regulation, cell differentiation, and development. The dysregulation of RBPs is associated with a variety of human diseases, including neurodegenerative disorders, autoimmune disorders, and human cancers. RBPs can alter gene expression and contribute to the pathogenesis of diseases. Therefore, RBPs represent prospective therapeutic targets for the treatment of these diseases. This Research Topic aims to gain a deeper understanding of the relationships between RBPs and non-coding RNAs (ncRNAs) to identify new potential targets for anti-cancer therapies.

Tian et al. constructed and validated an RBP-related model to forecast the outcome of hepatocellular cancer (HCC) patients. They created a risk categorization algorithm for HCC patients using data from two RBPs, BOP1 and EZH2. The risk score increased with higher diagnostic grade and clinical stage, and it was a significant predictor for the outcome. A nomogram that included both the risk score and the clinical stage gave improved predictive precision. This RBP-based prognostic model could be used to forecast HCC outcomes and aid in treatment decision-making.

Ma et al. observed that six RBPs were independently linked to the outcome of thyroid cancer patients. They confirmed the six-gene predictive model and developed a nomogram to guide therapeutic decision-making. Their experimental analysis showed that NUP153 and USB1 had a significant impact on cancer cell growth and migration. This research offers new insights into potential biomarkers for thyroid cancer, and the six-gene model may be useful in clinical practice.

Yang et al. showed that NAT10 is overexpressed in most tumors when compared to normal tissues. In several cancer types, elevated NAT10 transcript levels were substantially associated with negative outcomes. They found significant associations between NAT10 expression and immune infiltrates in HCC, such as CD8^+^ T cells, CD4^+^ T cells, B cells, endothelial cells, and fibroblasts, as well as immunologic marker gene sets. These findings suggest that NAT10 expression has a significant effect on the survival of pan-cancer patients and is possibly linked to tumor immune invasion. Thus, NAT10 might be a valuable cancer biomarker and future treatment target.

Xu et al. look into the levels and functional relevance of the lncRNA RP11-138J23.1 in gastric cancer (GC). The authors uncovered that RP11-138J23.1 expression was increased in GC tissues and revealed that suppressing RP11-138J23.1 greatly decreased GC cell proliferation and invasion. They also confirmed the tumor-promoting activity of RP11-138J23.1 using mouse models. Further research showed that the malignant impacts of RP11-138J23.1 were achieved by interacting with the HuR protein and raising the lifetime of *VAV3* mRNA. Overall, these results highlight that RP11-138J23.1 may be a potential treatment candidate for GC patients.

Yi et al. show that high levels of circ_0007534 are correlated with endometrial cancer (EC) development. In particular, increased circ_0007534 expression is associated with poor differentiation, late tumor stage, cancer invasion, and worse outcomes in EC patients. Cell assays demonstrate that circ_0007534 increases EC cell growth, migration, epithelial-mesenchymal transition (EMT), and paclitaxel resistance. Furthermore, circ_0007534 increases the progression of EC by serving as an inhibitor of microRNA-625 (miR-625), resulting in higher expression of ZEB2. Thus, their results emphasize that the circ_0007534/miR-625/ZEB2 pathway regulates EMT and paclitaxel resistance in EC. As a result, targeting this signaling pathway might be a hopeful approach for reversing paclitaxel resistance and attenuating EC cancer development.

Yan et al. review the most recent studies on the involvement of circRNAs in drug resistance in non-small cell lung cancer (NSCLC). CircRNAs mediate the tumor microenvironment and act as miRNA absorbers to influence articular signaling cascades and contribute to the formation of drug resistance. The authors suggest that targeting circRNAs could be a potential therapeutic strategy for suppressing drug resistance in NSCLC.

In a mini-review by Wu et al., the impact of the IGF2BP family on the development of head and neck squamous cell cancer (HNSCC) was examined. This review indicated that IGF2BPs could serve as new predictive indicators and therapeutic targets for HNSCC. However, more research is required to fully comprehend the mechanisms underlying the function of the IGF2BP family in HNSCC and identify potential clinical applications for targeting IGF2BPs.

The articles in this Research Topic discuss the therapeutic potential of RBPs and ncRNAs for various types of cancer. Multiple studies investigate the relationship between RBPs and ncRNAs and cancer outcomes, as well as their functions in cancer cell proliferation, migration, and drug resistance. These studies provide new insights into prospective biomarkers for cancer diagnosis and prognosis and serve as a foundation for the development of novel cancer treatment strategies. However, further research is needed to fully understand the mechanisms underlying the function of RBPs and ncRNAs in cancer and identify their clinical applications.

## Author contributions

All authors listed have made a substantial, direct, and intellectual contribution to the work and approved it for publication.

